# SARS-CoV-2 variant evolution in the United States: High accumulation of viral mutations over time likely through serial Founder Events and mutational bursts

**DOI:** 10.1371/journal.pone.0255169

**Published:** 2021-07-23

**Authors:** Rafail Nikolaos Tasakis, Georgios Samaras, Anna Jamison, Michelle Lee, Alexandra Paulus, Gabrielle Whitehouse, Laurent Verkoczy, F. Nina Papavasiliou, Marilyn Diaz

**Affiliations:** 1 Division of Immune Diversity, German Cancer Research Center (DKFZ), Heidelberg, Germany; 2 Faculty of Biosciences, University of Heidelberg, Heidelberg, Germany; 3 Program of Translational Medical Research, Medical Faculty Mannheim, University of Heidelberg, Heidelberg, Germany; 4 The Nightingale-Bamford School, New York, NY, United States of America; 5 Cornell University, Ithaca, NY, United States of America; 6 San Diego Biomedical Research Institute (SDBRI), San Diego, CA, United States of America; Centers for Disease Control and Prevention, UNITED STATES

## Abstract

Since the first case of COVID-19 in December 2019 in Wuhan, China, SARS-CoV-2 has spread worldwide and within a year and a half has caused 3.56 million deaths globally. With dramatically increasing infection numbers, and the arrival of new variants with increased infectivity, tracking the evolution of its genome is crucial for effectively controlling the pandemic and informing vaccine platform development. Our study explores evolution of SARS-CoV-2 in a representative cohort of sequences covering the entire genome in the United States, through all of 2020 and early 2021. Strikingly, we detected many accumulating Single Nucleotide Variations (SNVs) encoding amino acid changes in the SARS-CoV-2 genome, with a pattern indicative of RNA editing enzymes as major mutators of SARS-CoV-2 genomes. We report three major variants through October of 2020. These revealed 14 key mutations that were found in various combinations among 14 distinct predominant signatures. These signatures likely represent evolutionary lineages of SARS-CoV-2 in the U.S. and reveal clues to its evolution such as a mutational burst in the summer of 2020 likely leading to a homegrown new variant, and a trend towards higher mutational load among viral isolates, but with occasional mutation loss. The last quartile of 2020 revealed a concerning accumulation of mostly novel low frequency replacement mutations in the Spike protein, and a hypermutable glutamine residue near the putative furin cleavage site. Finally, end of the year data and 2021 revealed the gradual increase to prevalence of known variants of concern, particularly B.1.1.7, that have acquired additional Spike mutations. Overall, our results suggest that predominant viral genomes are dynamically evolving over time, with periods of mutational bursts and unabated mutation accumulation. This high level of existing variation, even at low frequencies and especially in the Spike-encoding region may become problematic when super-spreader events, akin to serial Founder Events in evolution, drive these rare mutations to prominence.

## Introduction

The Severe Acute Respiratory Syndrome Coronavirus 2 (SARS-CoV-2), which causes the Coronavirus disease 2019 (COVID-19), was first detected in December 2019 in Wuhan, China, when a number of severe pneumonia cases were reported [1]. By March 11^th^, 2020, the COVID-19 outbreak was classified as a pandemic by the World Health Organization (WHO) [2] and as of late May 2021, more than 171 million COVID-19 cases have been confirmed worldwide, while 3.56 million related deaths have been reported [3].

SARS-CoV-2 is an enveloped, single-stranded, positive-sense RNA virus and a member of the *betacoronavirus* genera, of the *Coronaviridae* family [4]. The viral envelope of SARS-CoV-2 consists of the membrane (M), envelope (E), nucleocapsid (N) and spike (S) proteins (encoded by the ORF5, ORF4 and ORF2 respectively), crucial components of the viral structure, but also necessary for the packaging of the viral RNA genome, and for viral infectivity [5]. The S protein (also known as the spike glycoprotein), is a major contributor to COVID-19’s pathogenesis and tropism, as it is responsible for SARS-CoV-2’s recognition, fusion and entrance into host cells. The infection process initiates when the Receptor Binding Domain (RBD; S1 subunit) of the S protein recognizes and binds the angiotensin-converting enzyme 2 (ACE2) receptor of the host, leading to fusion of the viral envelope with the cellular membrane thanks to a hydrophobic fusion peptide sequence found in the spike’s S2 subunit [6].

The entrance and subsequent release of the positive strand viral RNA genome in the host cell is followed directly by its translation into a variety of structural and non-structural proteins crucial for the viral life cycle [7, 8]. ORF1a and 1b are the first to be translated and encode the polyproteins pp1a and pp1b, which are cleaved by the papain-like protease (PL^pro^) and the chymotrypsin-like protease (also referred to as 3C-like protease; 3CL^pro^) [9]. This results in the production of 16 non-structural proteins (nsp1-11 from pp1a and nsp12-16 from pp1b) [9]. Together, these nsp proteins are necessary for the viral life cycle, as they regulate the assembly or are components of the Replication-Transcription Complex (RTC) [10]. Nsp1 “hijacks” the translational machinery of the host to prioritize viral protein expression [11], while Nsp2 modulates the host’s cell cycle progression, migration, differentiation, apoptosis, and mitochondrial biogenesis [12]. Nsp4 interacts with nsp3 and other host proteins to facilitate viral replication [5, 12], while the nsp6 protein induces membrane vesicles [13]. Nsp12 functions as an RNA-directed RNA polymerase (RdRp) and synthesizes the viral RNA with the help of the cofactors nsp7 and nsp8 [14]. Nsp14 is also part of the RTC by virtue of its function as a 3’-5’ exoribonuclease proofreader, among other functions [15]. Additional RTC nsp proteins are nsp9 (capable of binding to RNA), nsp10 (cofactor of nsp14 and nsp16), nsp13 (helicase and 5’ triphosphatase), nsp15 (with N7-methyltransferase function) and nsp16 (with 2’-O-methyltransferase function) [5, 16]. Once the RTC complex is established, it produces copies of negative-sense viral RNA, which are then used as templates for synthesis of the positive-sense genomic RNA (through an obligatory double stranded RNA intermediate [17]). These new copies of genomic RNA are either translated for the expression of new nonstructural proteins or are assembled into virions toward viral release [5]. Finally, the N protein binds to the newly synthesized positive-sense genomic RNA in the cytoplasm, forming the ribonucleocapsid, which along with the M, S and E proteins, are transported to the endoplasmic reticulum-Golgi intermediate compartment (ERGIC) for virion assembly. The virions exit the Golgi via budding and are released out of the cell through exocytosis [8].

All these ORFs encode components crucial to the SARS-CoV-2 life cycle. Genomic variants that alter the amino acid composition of any of these ORFs are of interest. Normally such variants would arise from polymerase-induced mutations during viral replication. However, SARS-CoV-2 (with a genome of ~30 kb) appears to mutate less frequently than viruses with smaller genomes [18], a feature attributed to nsp14, which possesses 3’-5’ exoribonuclease proofreading function that repairs some of the RdRp generated errors [15]. Indeed, the majority of single nucleotide variants detected in viral genomes (65% of documented mutations [19, 20]) are C-to-U and A-to-G base changes, a likely result of the action of RNA editing deaminases [21]. These enzymes of the APOBEC (Apolipoprotein B mRNA editing enzyme, catalytic polypeptide-like) and ADAR (Adenosine Deaminase Acting on RNA) families are normally referred to as anti-viral [22–24]. They target C’s in single stranded RNA (as is documented for APOBEC1 [25], APOBEC3A [26, 27] and possibly APOBEC3G [28]) or A’s in double stranded RNA (generated during viral genome replication–a perfect substrate for ADAR enzymes) to generate transition mutations (C-to-U and A-to-I, decoded as G) [23]. While RNA deamination in general (also referred to as RNA editing) is normally thought of as anti-viral, there is no reason why it cannot power viral evolution as well, and in fact, current data suggest it does so in SARS-CoV-2. Aside from single nucleotide substitutions, there is experimental evidence that, at least *in vitro*, this and earlier coronaviruses (e.g. SARS-1) are capable of recombination, through template strand switching [29].

Here, we have tracked the appearance of mutations in the SARS-CoV-2 genome through the first 15 months of the pandemic in the United States. Starting from aggregate mutational profiles, we derived a number of mutational signatures, representing distinct variants in 2020, which we have then tracked as they emerged across the U.S. in the course of the pandemic. We report an increase of variant emergence and mutations per variant with time, underscoring the need for continued mitigation even in the context of a successful vaccination strategy. Finally, we observed the emergence of variants of concern (VOC’s) gradually rising to prevalence from late 2020 into late March of 2021 including further evolved versions of the British variant of concern (B.1.1.7), underscoring the urgency of a dual strategy of mitigation and vaccination.

## Materials and methods

### The dataset

The NCBI SARS-CoV-2 Resources portal (https://www.ncbi.nlm.nih.gov/sars-cov-2/) was the source for all SARS-CoV-2 sequences employed in this study. To fulfill the criteria of nucleotide completeness (complete coverage), 62,211 viral isolate sequences were retrieved and isolated from human infections in the USA. Sequences retrieved by the time of analysis were isolated from infections reported between January 5^th^, 2020 and the end of March, 2021 (noted as “Collection Date” in the database). In our analyses, we considered the collection date as the most relevant parameter and interpreted our results according to this time frame.

### Variant calling and annotation

As the reference genome, we considered the one isolated from patient-zero in Wuhan, China (accession number NC_045512 in RefSeq). Alignments were performed with the software “VIRULIGN” in codon-corrected fashion, which reported the Single Nucleotide Variations (SNVs) compared to the reference genome [30]. Translation of SNVs to note amino acid changes were processed with an R (4.0.2) script, which applied the genetic code on reference sequence to display amino acid variation and thus highlight missense and silent mutations. Annotation of genomic variants with regards to regions in the viral genome (organized into ORFs) was performed employing NCBI RefSeq SARS-CoV-2 genome annotation, which is also publicly available in the NCBI SARS-CoV-2 Resources portal. Most variants and evolutionary signatures called throughout the dataset were visually inspected for validation of SNVs (and presumed amino acid changes). For further analysis and processing, different cut-off parameters were followed: as predominant variants in aggregate, we defined the missense mutations that are present in at least 10% of the genomes separately for 2020 and 2021. The same cut-off was used for nucleotide changes, but included both silent and missense mutations. For low frequency Spike mutations or add-on spike mutations in the variants of concern (VOC), we considered Spike missense mutations present in more than 0.1% of the genomes.

SARS-CoV-2 variants of concern (VOC) in the cohort of 62,211 sequences (viral isolates) were detected with the tool pangolin (https://github.com/cov-lineages/pangolin), according to the ‘cov-lineages.org’ lineage report [31] and the PANGO nomenclature [32].

### Mutational signatures analysis

We defined sequences with distinct combinations of the most frequently detected mutations in SARS-CoV-2 genomes as mutational signatures separately for 2020 and 2021 sequences. All unique combinations were called to build a reference of putative mutational signatures. We focused on those signatures that were found in more than 0.1% of the sequences. Time-scaled phylogenetic trees of the major signatures (>0.1%) was constructed with IQ-TREE 2 [33].

### Statistical analyses and visualization

All statistical analyses and visualizations were performed in R programming language (v. 4.0.2) employing functions from the R stats package, as well as the Tidyverse (v. 1.3.0) [34], pheatmap (v. 1.0.12), dendextent (v. 1.14.0) [35], msa [36], treeio [37] and ggtree [38].

## Results

### SNVs accumulate progressively with time throughout the SARS-CoV-2 genome

**Fig 1:**
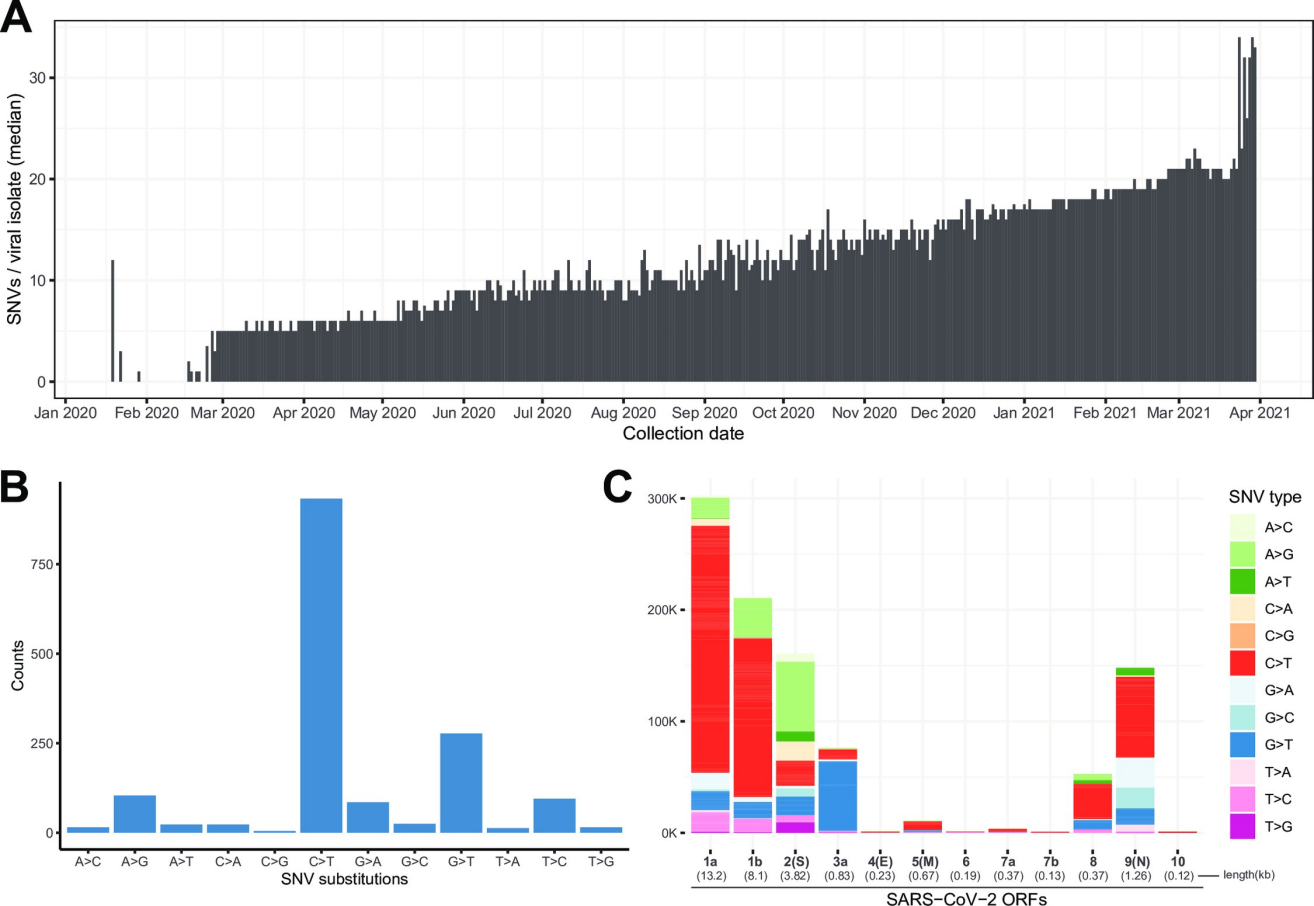
SARS-CoV-2 viral genomes accumulate specific sets of SNVs over time. (A) Frequency histogram showing the steady increase of SNVs called per viral isolate over time (Collection Date), indicating their accumulation in SARS-CoV-2 genomes. (B) Distribution of substitutions at unique SNVs. C>T and A>G substitutions have been previously associated with APOBEC and ADAR deaminase activities, on the SARS-CoV-2 ssRNA(+) genome or its dsRNA intermediate, respectively. (C) Graphical representation of SNV substitution profiles at various SARS-CoV-2 ORFs, illustrating intrinsic mutational bias for C>T dominating the mutation pattern in some ORF’s (i.e. 1a and 1b), but being masked (likely by selection) in other ORFs like ORF2 encoding Spike region. ORF lengths (kb) are given in parentheses across the x axis.

The SARS-CoV-2 isolates analyzed in this study were collected from infected American individuals between January 2020 and March of 2021 and encompass 62,211 genomes. The number of sequences per collection date and locations from which they are obtained are shown in S1 Fig. The number of SNVs per viral isolate increases progressively over time (Fig 1A), indicating the virus is not keeping a static genome during the course of the pandemic and is instead accumulating diversity. In the last months of analysis, there is a mutational jump indicating the arrival of VOC’s which are highly mutated compared to the original reference Wuhan strain.

The kinds of substitutions that characterize the aggregate viral SNV profile are predominantly C>T changes, with A>G, G>U and U>C also abundant but to a lesser extent among all mutations (Fig 1B). Unique (non-ancestral) synonymous changes revealed that C to T (U) and T (U) to C transitions are over-represented among synonymous changes (Table 1; synonymous changes typically represent roughly 1/3 of the mutations). Indeed, the large number of C to U mutations (by far the most common mutation among unique mutations) regardless of whether they generate a replacement or not, combined with their excess representation among synonymous changes, suggest the intrinsic signature of the mechanism generating mutations in SARS-CoV-2 involves the generation of C to U mutations with a secondary smaller bias for U to C. These base substitution patterns add to the increasing chorus in the literature that the APOBEC family of RNA editing enzymes may be contributing to SARS-CoV-2 diversity (not entirely surprising considering their known roles as antivirals [20, 21, 24]). In certain ORFs, C to U changes were predominant (Fig 1C) while others deviated from the intrinsic mutational signature such as ORF2 encoding spike, suggesting the intrinsic pattern may be masked by positive selection for other types of mutations in other ORF’s including ORF2. U to C transitions were over-represented among unique synonymous changes also pointing to intrinsic causes of mutation in SARS-CoV-2.

**Table 1:** Nucleotide substitution ratios of synonymous to non-synonymous changes among transtitions, G-to-A, A-to-G, C-to-U, U-to-C in 2020.

	G-to-A	A-to-G	C-to-U	U-to-C
Silent (S)	470	879	1446	1659
Missense (M)	1200	1310	1854	629
Ratio (S/M)	0.39	0.67	0.78	2.64

**Fig 2:**
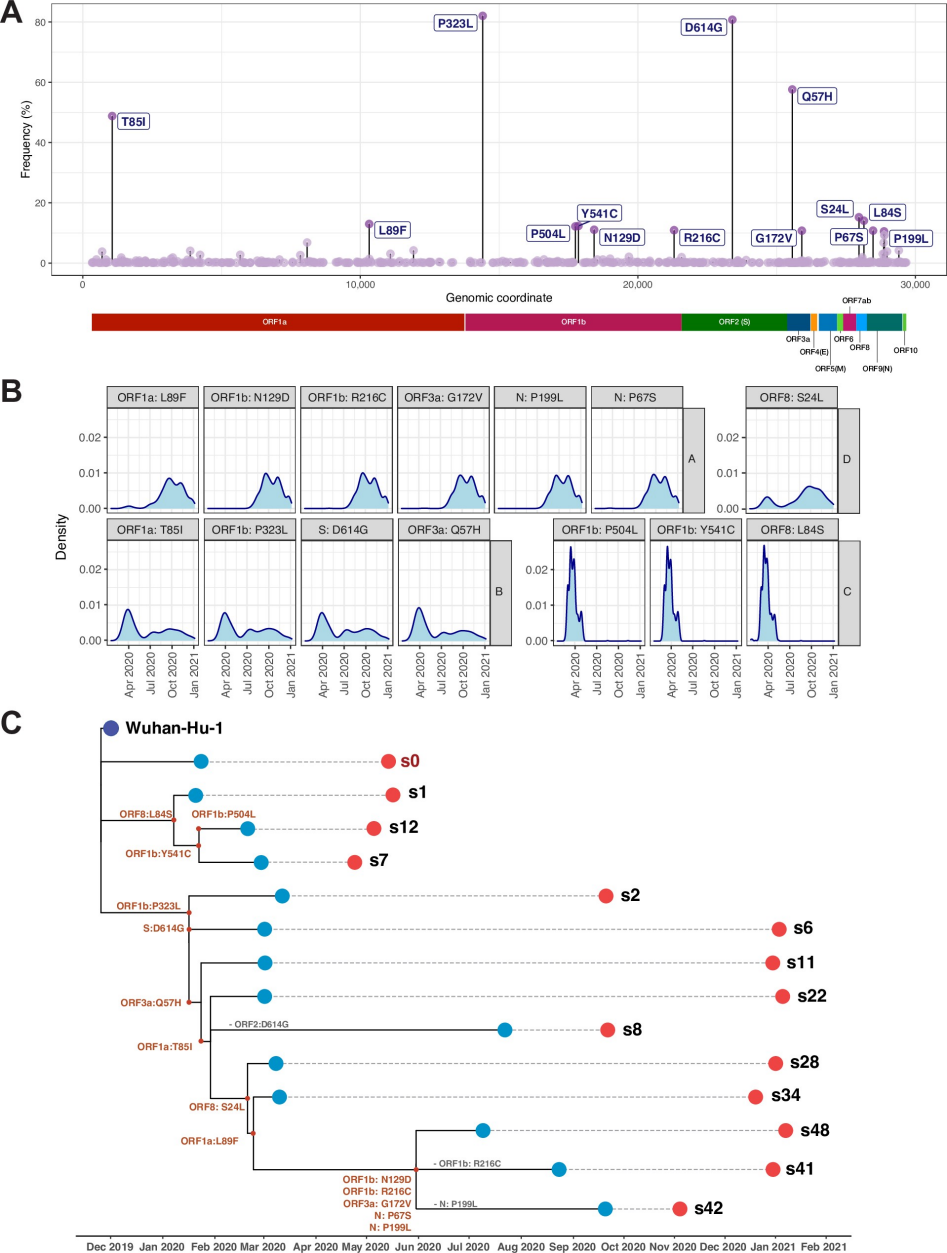
Accumulation of mutations in SARS-CoV-2 genomes and evolution of variants in 2020. (A) Dot plot representation of missense mutations identified in the SARS2-CoV-2 genome, of which fourteen were found in the most abundant SNVs, including Threonine-to-Isoleucine (T85I) and Proline-to-Leucine (P323L) changes in ORF1b (present in about 48.79% and 80.2% of the sequences, respectively) and the well-documented Aspartic-acid-to-Glycine (D614G) change in ORF2 (found in 80.76% of the sequences). A summary further detailing these predominant mutations is provided in the Table 2. (B) Density histograms (showing how the most common mutations from Fig 2A change over time), reveal that the most common mutations can be grouped into four distinct patterns (A-D); these mutational co-occurrences thus indicate the presence of at least three major variants. (C) Unique profiles of co-occurring mutational signatures from the dataset were employed to compile 48 sub-variant putative signatures (s1-s48; S2A Fig), distinct from the original Wuhan viral isolate (s0). 14 signatures and the s0, were found in more than 0.1% of the sequences. A time-scaled phylogenetic tree of those 14 subvariants and s0 (highlighted in red) reveals accumulation of mutations and more complex signatures with an acute burst of mutations in the summer of 2020 likely leading to a novel homegrown variant (s48). The first and last sequences by time profiled (per signature) are denoted with light blue and red dots respectively. The reference genome (Wuhan-Hu-1) is denoted with a dark purple dot. Gain of mutations in the clades is denoted with red letters for each specific mutation, while loss with grey. The most abundant signatures in the end of 2020 and early 2021 are s6, s22 and s48 (also shown in S2B Fig).

For our analysis, we focus on the evolution of the virus in 2020, as the predominance of VOC’s in 2021, particularly B.1.1.7, and the short period we have data for since its arrival, obscure any subtle evolutionary patterns important to our understanding of viral evolution dynamics. In our viral isolate cohort for 2020, fourteen specific missense mutations were found at high frequencies in the aggregate sequence data (Fig 2A; Table 2) suggesting they were under positive selection. Mutations that appear in more than 10% of the retrieved sequences and whose frequency over time profile suggest at least three major variants include the following:

1. in ORF1a, a Threonine-to-Isoleucine (T85I) change is present in 48.79% of the sequences, leading to a recoding effect in the Nsp2 protein (one of the first viral encoded proteins to initiate the viral life cycle; also described in [39]). Additionally, a Leucine-to-Phenylalanine (L89F) change occurs in 12.98% of sequences, recoding the peptidase C30. The frequency of this latter mutation follows a specific pattern (pattern A, Fig 2B), where it increases over time concurrently with other mutations of the same pattern, as mentioned below.
2. In ORF1b, a Proline-to-Leucine change (P323L in 82.03% of sequences) appears to be the most frequent mutation found in our cohort and has been previously also found in [40]. In the same ORF, Y541C and P504L represent recoding changes affecting the DNA/RNA helicase domain, and N129D and R216C represent amino acid changes in Nsp14 and Nsp16 respectively [41]. Not all of these mutations follow the same frequency patterns (for example P323L, P504L and Y541C, and R216C follow distinct patterns as shown in Fig 2B).
3. In ORF2, an Aspartic-acid-to-Glycine (D614G) change occurs in 80.76% of sequences (pattern B, Fig 2B) maps between the receptor-binding domain (RBD) and the spike’s S2 subunit. This change has been extensively noted in the literature as a variant associated with increased infectivity and appears to have originated in Europe [42].
4. In ORF3a, a Glutamine-to-Histidine (Q57H) mutation found in 57.62% of the sequences (pattern B, Fig 2B) is also found along with a G172V change [43] (pattern A, Fig 2B), both recoding the viroporin protein of SARS-CoV-2 [44].
5. Mutations in the Ig-like (ORF8) and Nucleocapsid (ORF9) proteins of SARS-CoV-2 have also been abundantly found: in the former an S24L change, which follows a unique frequency pattern (pattern D, Fig 2B), as well as an L84S change (pattern A, Fig 2B), both at about 15% frequency [45] and in the latter, a P199L change [46] and a P67S alteration, the latter which has not been previously documented, both found in about 10% of the sequences (with a frequency pattern A, Fig 2B).

**Table 2:** Summary of predominant mutations detected in SARS-CoV-2 genomes in 2020, indicating their nucleotide position (relative to the reference genome), the ORF they are located in, the associated amino acid change, the related protein that recoding may impact, and the frequency (% of sequences) at which they occur.

Change (Nucleotide)	ORF	Change (Protein)	Protein Function	% Frequency
C14408T	1b	P323L	RNA-dependent RNA polymerase	82.03%
A23403G	2 (S)	D614G	Spike protein; between RBD and S2	80.76%
G25563T	3a	Q57H	APA3 viroporin–accessory protein	57.62%
C1059T	1a	T85I	Nsp2	48.79%
C27964T	8	S24L	Ig-like protein	15.22%
T28144C	8	L84S	Ig-like protein	14.07%
C10319T	1a	L89F	Peptidase C30	12.98%
A17858G	1b	Y541C	DNA/RNA helicase domain	12.34%
C17747T	1b	P504L	DNA/RNA helicase domain	12.19%
A18424G	1b	N129D	Nsp14; 3’-5’ exonuclease	11.08%
C21304T	1b	R216C	Nsp16	10.93%
C28472T	9	P67S	Nucleocapsid	10.78%
G25907T	3a	G172V	Viroporin	10.76%
C28869T	9	P199L	Nucleocapsid	10.53%

The genomic variants presented in this table are the ones found in more than 10% of the sequences and annotated in Fig 2A. This table is restricted to mutations in 2020. Mutations predominant in Q1 2021 are mostly attributable to the arrival of B.1.1.7 (now Alpha). All Q1 2021 VOC’s are shown in Figs 4 and 5 and S4 and S5 Figs.

The identified patterns of specific mutations group with near-identical “frequency over time” profiles, suggesting at least three major variants (see below) were present in the United States at various time points in 2020 (Fig 2B and S2B Fig). Some of these mutations are found more frequently earlier in the pandemic (pattern C, Fig 2B), and thus correlate with the original Wuhan strain and its early derivatives.

### Mutational signatures over the SARS-CoV-2 genome suggest a combination of genetic drift and selection

From our sequence cohort of 2020, we determined all potentially distinct mutation combinations among sequences to get a sense of the evolution of SARS-CoV-2 in the United States. We found 48 distinct putative signatures (s0-s48) that ranged from extremely rare (1 genome) to frequent (in more than 10% of the genomes) (S2A Fig). We focused on those signatures that were present in more than 0.1% of the genomes (Fig 2C). Their prevalence as a function of time was also evaluated (Fig 2C, S2B Fig). Three major variants appear to have dominated the landscape in the US in 2020. These include: (a) the reference Wuhan sequence which disappeared as of June 2020, (b) the D to G clade (D614G) and various lower frequency but highly similar subvariants and (c) a group of signatures from that clade that appear to have acquired multiple mutations in a relatively short period of time in the summer of 2020 (involving at least 5 missense mutations (Fig 2C)).

Significant divergence from the original Wuhan strain is already apparent in mutational profiles of SARS-CoV-2 genomes collected between March and May 2020 (part of the 1^st^ wave). The net effect was that sequence diversity among viral isolates increased with time but diversity may have come in bursts like the one seen in the summer of 2020, leading to the s48 signature (likely a homegrown variant; Fig 2C). Intriguingly, one of the mutations that define s48, N6054D, appears to impact the proofreading activity of SARS-CoV-2 [41], raising the possibility that the mutational burst is associated with this mutation. However, an earlier mutation in the RNA polymerase domain in the G clade may have also resulted in increased mutagenesis and proliferation of variants (Fig 2C; P323L). These data clearly indicate the genome of SARS-CoV-2 is not static and can adapt through mutation.

### Multiple low frequency missense mutations of unknown consequence in Q4 of 2020 accumulated in the Spike, warranting close surveillance

**Fig 3:**
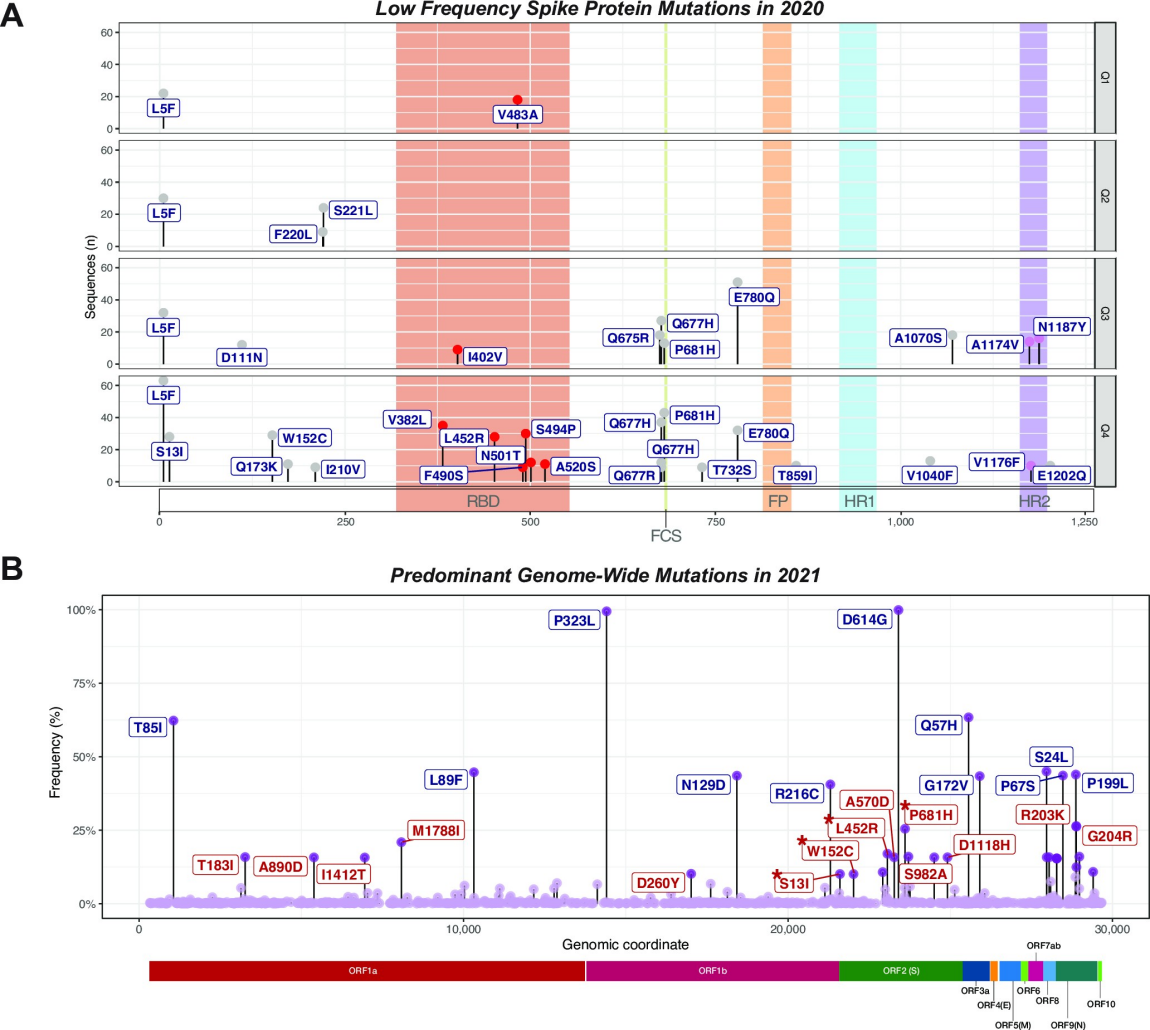
Some low frequency mutations in late 2020 rise to prevalence in 2021. (A) Dot plots showing accumulation of multiple LFSMs (>0.1% of cohort) in ORF2 over time in 2020. The amino acid position per LSFM is shown at the bottom, while quartiles, from the first (Q1) till the last (Q4) are denoted on the right. Mutations in Spike domains are further denoted by shaded areas. A detailed list of all LFSM in 2020 is provided in S3 File. (B) Dot plot representation of missense mutations identified in the SARS2-CoV-2 genome in 2021 and revealing VOC’s arrival. 34 mutations were found in at least 10% of the genomes (dark purple), most of which are common with the ones identified in 2020 (Fig 2A, Table 1), including P323L and D614G, which are present in 100% of the genomes analyzed in 2021. A number of newly predominant mutations in the Spike appeared in 2021 (red labels), include some previously found in 2020 Q4 (red labels with asterisk) as well as novel ones originating from VOC’s. Not all mutations are named but the main ones are S13I, A570D, P681H, W152C, S982A and D1118H. The complete set of mutations found in more than 10% of the sequences in 2021 is shown in S3A Fig.

Additional mutations were found in the Spike region that were found at low frequencies in late 2020 (Low Frequency Spike Mutations herein designated LFSMs; entire list shown in Fig 3A), but present in at least 0.1% of sequences (a cutoff selected to minimize sequencing error contribution to the analysis). The consequences of such LFSMs to infectivity, severity of disease, or response to vaccination remain unknown. They include: L5F (163 genomes), E780Q (83 genomes), P681H (74 genomes) and Q677H (68 genomes; see below), as well as over 20 additional, mostly unidentified amino acid replacing mutations (Fig 3A). None of these LFSMs have been identified as problematic to date. Strikingly, LFSMs seem to be increasing over time as more and more mutations accumulate in the 4^th^ quartile—while only a couple have been lost likely from genetic drift (Fig 3A). Amongst all LFSMs, six were found in the receptor binding domain (RBD) (Fig 3A) and include (with number of genomes in parentheses): V382L (35), L452R (28), F490S (9), S494P (30), N501T (12), and A520S (11), all which may have consequences for binding affinities to the ACE2 receptor in human cells, infectivity and/or response to vaccines developed to trigger antibody responses to the RBD of earlier strains. Moreover, we identified two LFSMs with different amino acid substitutions at Q677: Q677R (A23592G) and Q677H (through two different point mutations -G23593T and G23593C) which are very close to the furin cleavage domain. This hypermutability at Q677 suggests that it has been under strong selection. A series of then novel LFSMs were detected in Q4 of 2020 and included (with number of genomes in parentheses): I210V (9) T732S (9), E780Q (32), T859I (10), V1040F (13), V1176F (10), and E1202Q (10). Finally, it is important to note that such LSFMs are still present within the US population (see below), and many are potentially one super-spreader event away from prominence, which could thus lead to problematic new mutations or variants.

### Appearance of SARS-CoV-2 variants of concern late in 2020 and rise to prevalence in 2021

The functional consequences of variant evolution are most obvious in the context of Spike protein, as mutations in Spike could impact receptor recognition and infectivity (as well as alter antibody binding and thus lead to immune evasion). Such Spike variants will thus herein be denoted as “variants of concern” (VOCs). One of the first VOCs was the D614G mutation (clade G) [42], which is now found in the vast majority of SARS-CoV-2 genomes (including all genomes recently annotated as novel variants of concern, such as B.1.1.7). Indeed, D614G is present in more than 80% of the sequences in our cohort in aggregate (Fig 2A, Table 2), and virtually all sequences from after the 2^nd^ quartile of 2020 (Q2) have this mutation. In addition to the previously described VOCs, we detected multiple isolates with a H69/V70 deletion in 2020, with some having an additional deletion at V143/Y144 suggesting the arrival of the B.1.1.7 lineage according to [32] in late 2020. Others, then rare, lineages carry the H69/V70 deletion together with a handful of other mutations, matching the B.1.375 variant. We also detected a K1191N mutation in the HR2 domain of B.1.1.7. This mutation has been found in at least one other variant in Bangladesh, suggesting this may be another problematic recurrent mutation under positive selection [47]. These findings highlight the ongoing diversification of the Spike region.

Strikingly, the arrival of the VOC’s in late 2020 Q4 and in 2021, is reflected in the sudden appearance of prevalent novel mutations in Spike in 2021 (Fig 3B). Additionally, a number of newly predominant mutations in Spike in 2021 were previously found in 2020 Q4 at low frequency (Fig 3B: red labels with asterisk). The complete set of predominant Spike mutations in 2021 (found in more than 10% of the sequences) is shown in Fig 3B and S3A Fig. A figure listing the defining mutations for VOC’s is in S3B Fig).

### Dramatic change in SARS-CoV-2 signatures across US states from 2020 to 2021 indicate ongoing evolution and the arrival of B.1.1.7 and other variants of concern

**Fig 4:**
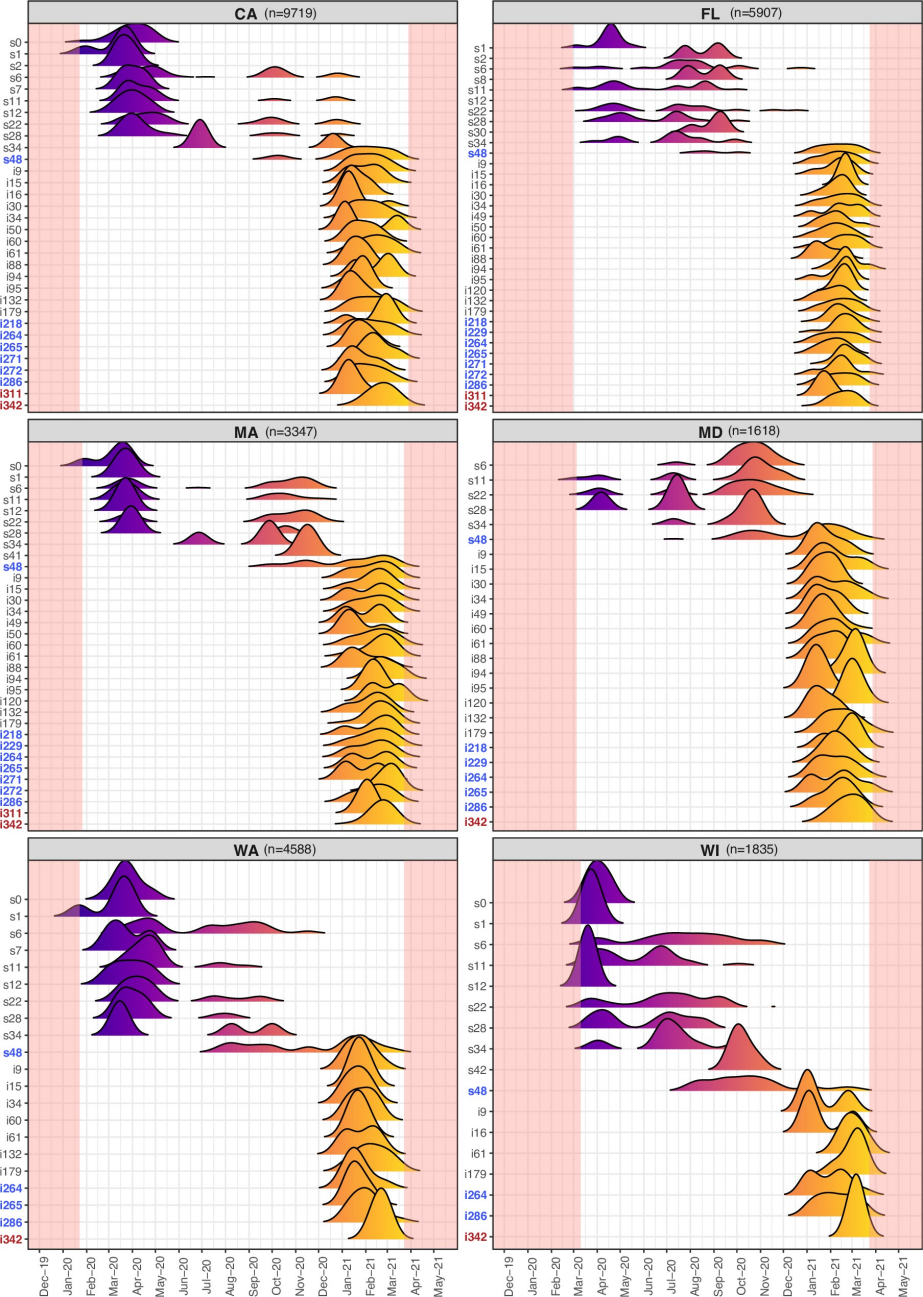
SARS-CoV-2 viral isolate signature frequencies change over time, but with different patterns across states, showing dynamic evolution by mutation, drift, selection and migration. State-specific ridgeline plots indicate the density of each signature (y axis) over collection date (x axis). In each plot, peak colors gradually change to highlight transition in time (x axis), with the pink-shaded areas corresponding to periods of time where data was not available. States shown were selected only on abundance of sequence data throughout the year (n, number of viral genomes per state). Of note, the reference strain s0 is virtually absent by June 2020, while signature s48 is common from July 2020 through the end of the first quartile of 2021. Several new signatures found predominantly in 2021 (in more than 0.1% of the genomes) reveal a new and complex mutational profile, with a number of them being related to B.1.2 (blue labels), or B.1.1.7 (red labels) lineages (which were introduced by migration), as well as of other VOC lineages (S3A, S4 and S5 Figs).

Significant divergence from the original Wuhan strain is already apparent in mutational profiles of SARS- CoV-2 genomes collected between March and May 2020 (part of the 1st wave) and in 2021, the new strains dominate the landscape (Fig 4). In 2020, several mutational signatures become dominant over time, a pattern specific to some states and anchored by the well-known D614G mutation. For example, in California, a diverse set of signatures is present early on, but by the end of 2020, s6, s11, s22, s28 and s48 dominate (Fig 4). These signatures are known as part of the B.1.2 clade. Of note, some signatures are state-specific such as s41 in MA, and s42 in WI, both very similar to the now ubiquitous s48 but with the apparent loss of a single mutation in that lineage (Fig 4), likely through genetic drift. The net effect is that sequence diversity among viral isolates increased with time but diversity may have come in bursts, as was seen in the summer of 2020 leading to the s48 signature, likely a homegrown variant. These data from 2020 clearly indicate the genome of SARS-CoV-2 is not static and can adapt readily through mutation. In Q1 of 2021, the pandemic in the US was characterized by the arrival of a completely different set of signatures across all states, in addition to a further diversified B.1.2 lineage and derivatives of B.1.1.7 (Fig 4). Analysis of all lineages and signatures identified in 2021 is provided in S3A, S4, and S5 Figs describe their lineage origins.

### VOC’s continue to mutate in the United States in 2021

**Fig 5:**
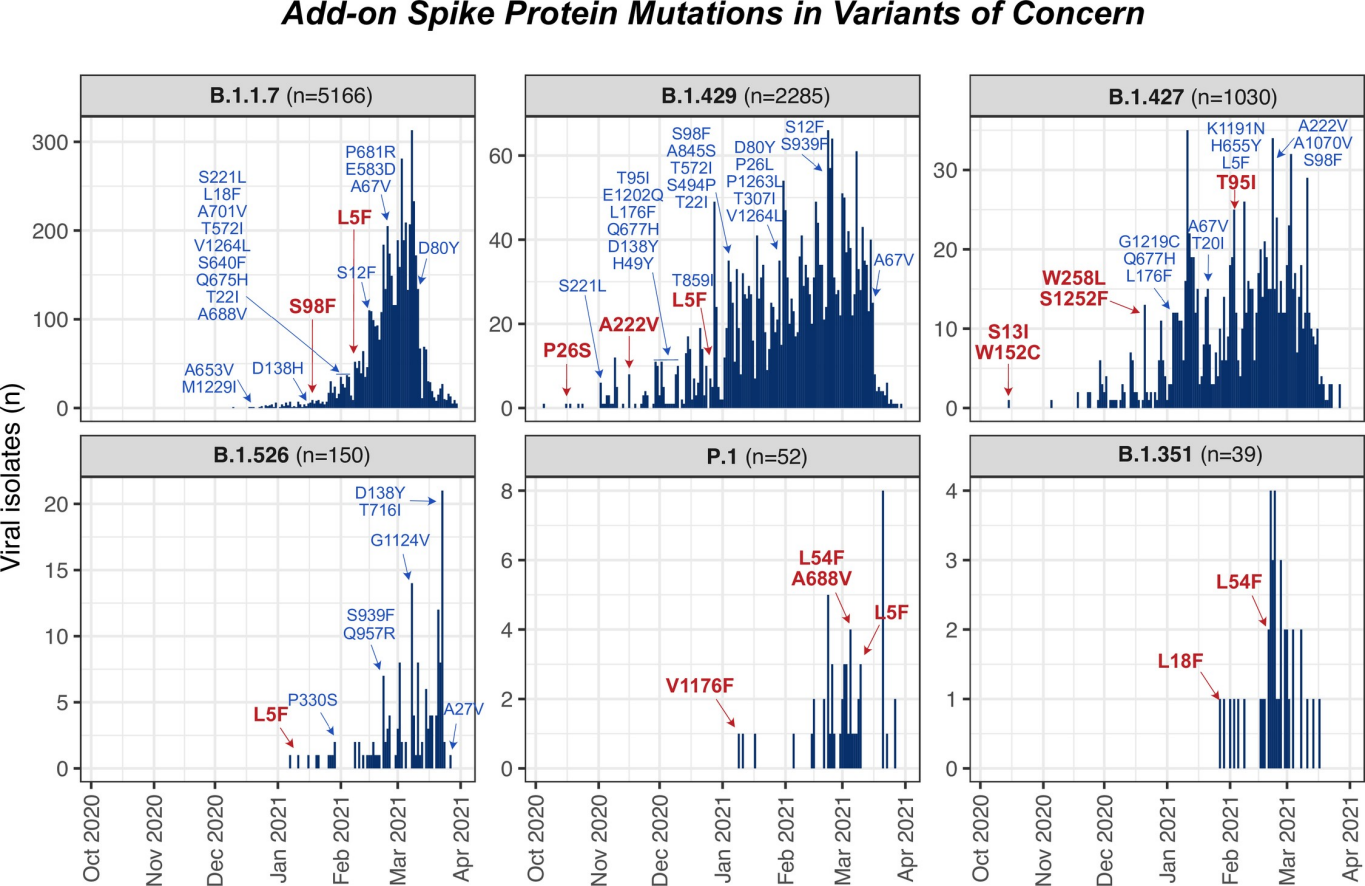
Variants of concern emerging in the United States include novel “add-on” mutations in key Spike protein functional domains. 6 variants of concern (VOCs), B.1.1.7, B1.351, B.1.427, B.1.429 and P.1, were detected in the cohort of sequences we explored, primarily in the last quartile of 2020 and the first of 2021. Clearly VOC genomes are accumulating a diverse set of new spike mutations, in addition to their defining mutations (S3B Fig). Bar plots show the number of sequences (n) of the 6 different VOCs per collection date. Red labels annotate the introduction of new spike mutations in the genomes of the reported VOCs in time in more than 1% of the respective VOC, while less frequent mutations (>0.1% of genomes) are shown in light blue labels. Of note, the most common mutation is L5F, which, in being present robustly throughout time and across variants, suggests it may be a recurrent mutation. Interestingly, other mutations previously seen as LFSMs in 2020 (Fig 3A), including Q677H or Q677R, T859I, E1202Q, V1040F, V1176F are also present in distinct VOCs, suggesting multiple recurrent mutations that may reflect mutational or selection bias.

Concerning but not surprising data indicate that VOC’s have continued to acquire new mutations in 2021, making the possibility of vaccine escape mutants more likely (Fig 5). Several of these mutations seem to converge across lineages suggesting they are recurring and are under positive selection (see L5F, and the hypermutable region around the furin cleavage site such as mutation Q677H; Fig 5). Most of the mutations occurred in a relatively small number of isolated VOC genomes (see S3 File for table with information for mutations and number of genomes). However, as seen for low frequency variants in 2020 Q4, super-spreader events can help increase low frequency mutations to prevalence. However, unlike in 2020, a vigorous vaccination program may be keeping divergent B.1.1.7 genomes in check. Genomic surveillance of this and other variants is critical to guard against the possibility that an escape clade, now lurking in the population, might expand and take over, as mitigation measures are relaxed.

## Discussion

Among positive strand RNA viruses, the genome of SARS-CoV-2 has been thought to be remarkably stable–in part because it has proofreading functionality during RNA synthesis—a function carried out by nsp14 [15]. However, this notion of stability has come under scrutiny with the emergence of multiple variants, some threatening the effectiveness of vaccines, and many coinciding in convergently acquired spike mutations [48]. Indeed, though lacking the diversity seen in HIV-1 variants [49], SARS-CoV-2 is fully capable of acquiring mutations that enhance its ability to spread and evade immune responses.

In this study, we aimed to examine SARS-CoV-2 variants in the United States during the first year of the pandemic. We were interested in sampling the existing variation, how variant frequency changes over time and across states and finally, in the potential identification of either new variants or novel mutations in pre-existing variants that have arrived from other parts of the world. We also examined whether the pattern of mutations, particularly among synonymous sites, could provide a clue as to how, despite its proofreading exoribonuclease activity, SARS-CoV-2 has accumulated significant genetic variation.

For this study, we obtained 62,211 full length sequences from Covid19 patients from January 2020 through April of 2021 from 42 US states. It is important to note that a majority of the data was obtained from a handful of states (California, Florida, New York, Maryland, Massachusetts, Minnesota, Virginia, Wisconsin, and Washington (S1B Fig)), likely a combination of available genome surveillance programs, and rates of infection in those states. In 2020, we identified several distinct variants (Fig 2C, S2A Fig) that can be categorized as follows: **1)** The original Wuhan strain and a few descendants with minor changes. This strain lacks the D614G change that emerged in Europe early in the pandemic (G-clade). The reference strain and its minor subvariants appear to have been lost in most states by early to mid-summer (Fig 3); **2)** Two versions of the G-clade European strain defined by the acquisition, in an intermediate within the clade, of multiple mutations within a short period of time in the summer of 2020, leading to the now predominant likely homegrown variant s48 signature (Fig 2C). Our analysis is not compatible with the notion that the burst of mutations originated from a recombination event; rather these mutations appear to arise from the acquisition of multiple single base substitutions that increased in frequency in the population relatively quickly, likely through serial Founder Events. However, a few examples of lone mutations shared across variants suggest the possibility of recombination or convergent evolution. Bursts of mutations may also originate from patients with persistent infection despite treatment with convalescent plasma where pressure for immune escape variants may be prolonged and intense [50, 51].

### Evolution of SARS-CoV-2 in early months of the pandemic in the United States

Strikingly, the main variants in the US in 2020 accumulated an increasing number of mutations over time (Figs 1A, 2B, 2C, 3, S2B Fig). This underscores the fact that with uncontrolled infection, the appearance of new mutations will increase. Of particular concern is our finding that over the last year in the United States, over 20 amino acid replacing mutations arose in the Spike protein that have not been identified yet as problematic, many still remaining in the population but currently at low frequencies (less than 1%, Fig 4C). Typically, mutations need to reach non-trivial frequency levels to survive genetic drift and loss from the population. However, the number of amino acid replacements impacting ORF2 encoding Spike seems to be increasing over time, with little corresponding loss of variation through drift (Fig 4C). This low frequency variation in Spike is of concern because of the number of super-spreader events in the US population leading to serial Founder Events that can increase the frequency of these rare mutations. Variation reaching non-trivial frequencies can then be subjected to positive selection in viral evolution from host immune escape variants in immunocompromised patients receiving convalescent plasma [50, 51], or in inadequately vaccinated individuals (e.g. having only received a single dose of a two-dose vaccine regiment). Competition between variants may also be a source of selection and may be the reason the D614G variant became the dominant form in most countries [52, 53].

Super-spreader events may effectively work as Founder Events in this pandemic. In Founder Events, where a few organisms initiate a new population, typically most genetic variation is lost [54]. However, multiple or serial Founder Events originating from a population can potentiate the generation of new species (or variants in this case) by providing a mechanism for rare mutations to quickly increase their frequency [55]. Therefore, in considering the generation of diversity of SARS-CoV-2, superspreading events is another mechanism, besides mutation, where the virus can effectively increase its diversity over the population. This provides a compelling reason to adhere to strict mitigation controls especially in the context of gatherings among unvaccinated people or when a new variant emerges in the context of poor vaccine coverage. It would thus seem prudent to control super-spreader events until the pandemic and variants are controlled across the world. Premature relaxing of mitigation measures while multiple countries struggle with new variants will likely prove to be consequential in the United States and the world at large.

### Mutations in Spike and other regions that may have clinical relevance

Deservedly, a lot of attention has been given to variations in the ORF encoding spike protein (Orf2), since any immune escape mutants are likely to arise particularly (though not exclusively) within the receptor binding domain of Spike, that interacts with the ACE2 receptor [56]. In 2020, we detected rare instances of the B.1.1.7 variant (3 cases in November in California and Florida) and 10 of the B.1.375 variant, recently identified as having the H69/V70 deletion similar to B.1.1.7 but lacking most of the other distinguishing mutations of B.1.1.7 [57]. This novel lineage (B.1.375) is another example of the H69/V70 deletion been found in independent variants, suggesting it evolved convergently multiple times in SARS-CoV-2 variants (as recognized by others [58]) even among different species [59]. Recent models suggest that the H69/V70 deletion may be a gateway to more variation, as it may provide increased flexibility of the receptor binding domain to accommodate mutations in the ACE2 receptor among individuals and/or species [56], but this remains speculative. By April of 2021, B.1.1.7 rose to high prevalence in the United States while other non-B.1.2 lineage variants remained at very low frequencies. This is likely to change in the upcoming winter season when new B.1.2 variants that are partially (or even fully resistant) to current vaccines gain prevalence in the US over B.1.1.7, barring new updated vaccines. Of note, our 2021 data shows all the VOC’s are continually acquiring new mutations here in the US.

In addition to variants within the ORF encoding spike protein (Orf2), we and others found significant variation in other Orfs such as Orf1a and 1b, including a 15bp deletion in the region encoding NSP1, previously identified in Japan [60]. The functional relevance of this variation is less clear but cannot be ignored as it may impact the virus’ ability to replicate, infect other cells once inside the host, and even modulate the host immune response (as has been observed for NSP1 [60]). In addition, a mutation in the RNA polymerase domain in the D to G clade, seems to coincide with mutational bursts raising the possibility of increased mutagenesis by the RNA polymerase. Therefore, non-spike mutations may impact the generation of new variants through increased mutagenesis, the severity of the disease, and potentially the spread of the virus by increasing its efficiency in hijacking host cells and lowering the viral load threshold required to establish infection. Genome surveillance programs should not ignore mutations outside the Spike region, for these reasons.

### Mutational pattern in SARS-CoV-2 may reveal intrinsic cause of genetic diversity

Because the SARS-CoV-2 genome is not as stable as initially thought despite its proofreading activity, we examined the pattern of mutation among synonymous changes throughout the SARS-CoV-2 genomes to determine the intrinsic mutational signatures, a clue to mechanism. Among synonymous mutations, we found that C-to-U and U-to-C transitions were abundant and among all mutations, C-to-U changes were dominant. One source of C-to-U mutations in RNA is the APOBEC family of RNA editing enzymes, some with anti-viral properties known to deliberately attack the genomes or RNA viruses, such as in the case of Apobec3G and HIV [61]. The less frequent U-to-C mutations could also be due to RNA modification events occurring on uracil, and decoded as cytosine; modifications that could result in such a profile could include thiolation (e.g. 4-thio-uridine) or aminocarboxypropylation (e.g. acp3U) events. Though these modifications have not yet been reported to occur on mRNA, both can occur on tRNA [62, 63]. Finally, A-to-G events are also evident and the likely result of adenosine deamination to inosine, decoded as guanosine, which is catalyzed by ADAR proteins, whose preference for dsRNA targets could attract them to double-stranded RNA intermediates of the viral replication process (the prominent G clade mutation (D614G) may be the outcome of an A-to-I deamination event at position A23403).

While we are not the first to make the observation that the SARS-CoV-2 genome is a target of modification enzymes [21, 64–66], but the fact that: (a) many of the mutations giving rise to VOC’s could be explained by such modification events, together with the fact that (b) known modification enzyme microsequence biases match mutations in VOC’s, lead us to speculate that RNA modifications are a major source of targeted mutagenesis of the SARS-CoV-2 genome. This would explain why emerging variants (like B.1.1.7), rather than diverging in sequence, appear to be acquiring mutations common to unrelated strains (e.g. the new acquisition of the E484K (**G**AA = >**A**AA mutation first defined as concerning in the unrelated 501Y.V2 variant [67], which could be attributed to a modification such as m1G, which can be decoded as A [68]). This does not eliminate the potential for recombination as another source of variation, as seen often in coronaviruses [29]; however we did not detect evidence of recombination events in the sequences we queried here.

### In 2021, VOC’s redefined the pandemic in the US, particularly B.1.1.7

While several B.1.2-like signatures persisted into 2021 (along with the California variants), B.1.1.7 rose to prevalence as predicted from its previous trajectory in the U.K. Other VOC’s seemed to have been kept at low frequency by the predominance of B.1.1.7. This is good news for now, as most available vaccines are highly effective against this strain. However, our data also indicate the acquisition of multiple mutations in B.1.1.7 in the United States albeit currently at very low frequencies. Based on the 2020 data, these new mutations could be problematic as they may rise to prevalence through super-spreader events; but for now, it appears this was prevented by the current and ongoing aggressive vaccination campaign.

### Potential for divergent VOC’s to lead to ADE

Another potentially troubling implication of the emergence of new Spike variants, particularly in VOC’s, is the potential for the concurrent development of neutralizing antibodies that are optimal for the original strains but not for the new strains, which may lead to the development of antibody-dependent enhancement (ADE) or antibody dependent inflammation (ADI) [69] documented for other viruses [70]. Fortunately, ADE has not emerged explicitly as a substantial concern with SARS-CoV-2 [71, 72], although suspects are MIS-C or MIS-A, the Kawasaki-like syndromes associated with COVID-19 infection and re-infection both in children and more recently also in adults [73, 74].

### Study limitation

One limitation of this study is that the pandemic is constantly changing making it difficult to report the most up to date mutations in the United States. In fact, at the time of this publication, a new variant, (B.1.617, now termed “Delta”) is starting to take a hold on the US population. However, the concepts demonstrating the pivotal role that superspreading events have in perpetuating the pandemic, increasing the frequency of new rare mutants, and potentially generating new variants of concern are forces that remain within areas of the country with low vaccination rates and underscore the importance of continuing mitigation measures including limiting large indoor events in these areas.

## Conclusion

From a public health perspective, this study underscores the critical importance of mitigating infection levels and particularly, super-spreader events, as potential generators of high frequency novel variants from the very low frequency existing and increasing Spike mutational pool. Indeed, the finding of over 20 spike variants at low frequencies in the population of the U.S., in late 2020 is concerning as this “lurking” genetic variation can quickly emerge as novel variants through super-spreader, Founder-like events in an expansion process similar to genetic surfing [75, 76]. All these considerations require, in addition to recommended mitigation efforts such as social distancing and mask wearing, that large scale vaccination be in accordance to the schedules used in the clinical trials leading to federal agency approval. Further supporting this, are reports of problematic variants arising within individual immunocompromised patients treated with convalescent sera [50] as these escape mutants can clearly arise when subjected to low levels of anti-Spike protein antibody. This, and our finding of potential “lurking” low frequency variants already within the population, dictate that selection against this virus through vaccination be strong and that “taking the foot off the pedal” (for example with low second shot compliance) can allow this existing variation to give rise to novel escape variants. The rise of VOC’s in 2021 in the US, and their further accumulation of mutations could have been catastrophic without vaccination. However, it would be naïve to assume this pandemic is over. B1.1.7 appears to have kept other VOC’s in check in the US thus far, but that will change as it is highly susceptible to existing vaccines. Will this give other VOC’s for which vaccines are less efficacious a chance to spread in late Fall, as mitigation efforts are relaxed? One concerning VOC is the B.1.617 variant (the Delta variant), which is spreading in the UK among unvaccinated people (and in China, Seychelles and Mongolia, among people vaccinated with adenorival vaccines that might be less effective against it). The relative success in efforts to develop and implement multivalent vaccines that include VOC’s versus those to control the pandemic in other parts of the world will be critical. Therefore, it would most likely be prudent in the late Fall to re-enforce indoor masking and limit large gatherings in the US to prevent a new surge of resistant VOC’s before a new generation of vaccines can, again, contain the pandemic.

## Supporting information

S1 Fig Chronological and geographical sampling information. (A) Bar charts showing the number of sequences per collection date. (B) Number of sequences per state, shown in the United States map with color scale ranging from blue (low) to red (high).
(TIF)

S2 Fig Associated information with Figs 2 and 3. (A) Heatmap summarizing all the putative signatures (columns) built by the unique combination of dominant mutations (rows). Signatures are ranked from left to right by the sequence number they were found in (red labels below x axis). The first 15 signatures that are found in 0.1% of the sequences (>8) or more were considered for the mutational signature analysis. Presence or absence of mutation in each signature is denoted with blue or light yellow respectively. (B) Signatures that were found in more than 0.1% of the viral isolates in aggregate were further explored in the context of time from emergence till early 2021. Viral isolates that were profiled with those signatures (s0, s1-2, s6-8, s11-12, s22, s28, s34, s41-42, s48), were ordered by collection date in the columns of the heatmap from left to right. The heatmap visualizes the % occurrence (light yellow to dark blue scale) of each signature per collection date in the cohort of sequences. Column annotation (bottom of the heatmap) denotes the different quartiles (Q1-Q4) of calendar year 2020 (very few entries from Q1 of 2021 are shown) in which the sequences were collected. The non-variant SARS-CoV-2 (s0) is present primarily in the in Q1 up to mid-Q2 of 2020. While a diverse set of signatures appears in the USA from the start of the pandemic onward, subvariants currently circulating in the American population are variations of s48, s22 and s6 (with some variation per state).
(TIF)

S3 Fig Associated information with Figs 3B, 4 and 5. (A) Heatmap summarizing all the putative signatures (columns) built by the unique combination of dominant mutations (rows) in 2021. Signatures are ranked from left to right by the sequence number they were found in (red labels below x axis). The signature s48, which emerged in 2020, was found in 5838 genomes in 2021, which ranks it the top signature also for 2021. Furthermore, additional signatures of the same lineage (B.1.2) emerged, likely from s48 (S4 and S5 Figs) in 2021. Furthermore, variants of concern, such as B.1.1.7, are responsible for an introduction of new signatures in 2021 (S4 and S5 Figs). (B) Schematic representation of the defining spike mutations or deletions (ΔH69/V70 and Δ144) for the different variants of concern detected in this study (Fig 5). The different domains of the Spike protein noted are RBD/receptor binding domain (red), FCS/furin cleavage site (green), FP/fusion peptide (orange), HR1/heptad repeat region-1 (turquoise) and HR2/heptad repeat region-2 (violet).
(TIF)

S4 Fig Associated information with Fig 4. The signature s48, first detected in July 2020 (Fig 2C) is still present through the end of March 2021 and in fact is the top signature with the most genomes it has been found in (n = 5838). With the addition of several more mutations found predominant in 2021, we called unique profiles of co-occurring mutational signatures compiling 348 additional signatures (i1-i348; S3A Fig) distinct from the original Wuhan viral isolate (s0). Only the ones present in 0.01% genomes were considered for further analysis. In the first quartile of 2021 the lineage that profiles most of the genomes is B.1.2 (12651 genomes), followed by the lineage of the variant of concern B.1.1.7 (5166 genomes). Different signatures showed specificity for the aforementioned linages and therefore time-scaled phylogenetic trees were constructed separately for data collected in 2021, rooted to the respective variant genome. The founding genome for B.1.2 in the cohort of sequences was signature s48 (purple node and root) which is still present throughout the first quartile of 2021 and has further expanded to other signatures through loss (grey labels) and gain (red labels) of its original mutation profile (S2A Fig). For B.1.1.7 lineage, the first sequence detected, with the defining mutations of B.1.1.7 was the signature i342, which through mutation loss and gain has given additional related signatures.
(TIF)

S5 Fig Associated information with Fig 4. A number of variants of concern (P.1, B.1.351, B.1.526, B.1.429, B.1.427, B.1.1.7) were detected primarily in 2021, while genomes of the B.1.2 lineage remained abundant. This network shows the different versions of signatures detected in 2021 (>0.01% of the genomes) in lineages of the different variants of concern. The thicker the connection is, the higher the number of genomes were found for that lineage. The number of genomes profiled with the different signatures shown in here can be found in S3A Fig.
(TIF)

S1 File Robust list of mutations (silent and missense) detected in the cohort of sequences in 2020 and 2021. Information related to Figs 2A and 3B.
(XLSX)

S2 File Low Frequency Spike Mutations (LFSM) detected in the quartiles (Q1-4) of 2020. Information related to Fig 3A.
(CSV)

S3 File Robust list of co-occuring add-on mutations in the variants of concern. Information related to Fig 5.
(CSV)
