# Supplementary figures and images for: SARS-CoV-2 variant evolution in the United States: High accumulation of viral mutations over time likely through serial Founder Events and mutational bursts
Source: PLoS One. 2021 Jul 23;16(7):e0255169. doi: 10.1371/journal.pone.0255169 (PMC8301627; doi:10.1371/journal.pone.0255169)

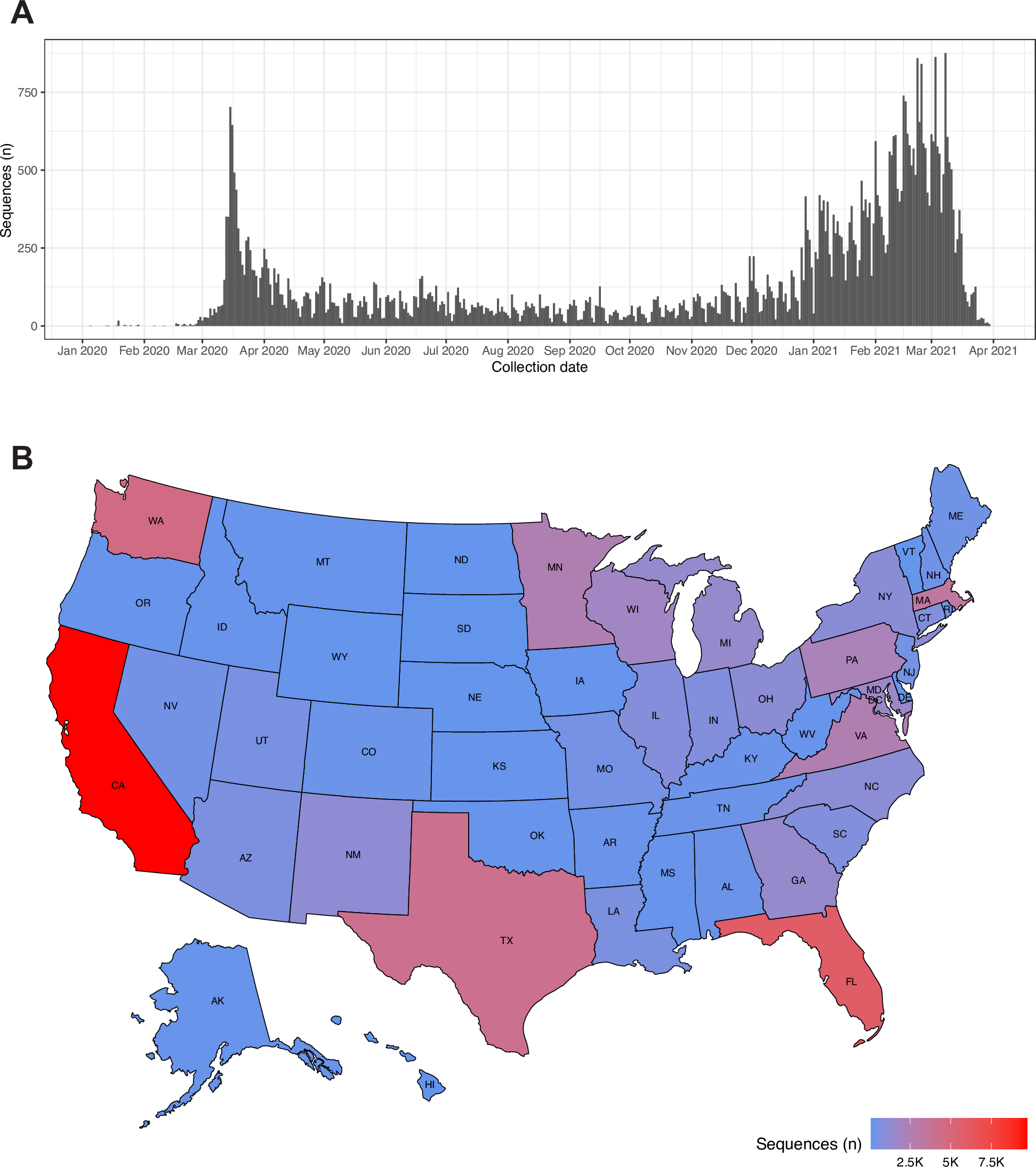

Supplement: S1 Fig — (A) Bar charts showing the number of sequences per collection date. (B) Number of sequences per state, shown in the United States map with color scale ranging from blue (low) to red (high). (TIF) [file pone.0255169.s001.tif]

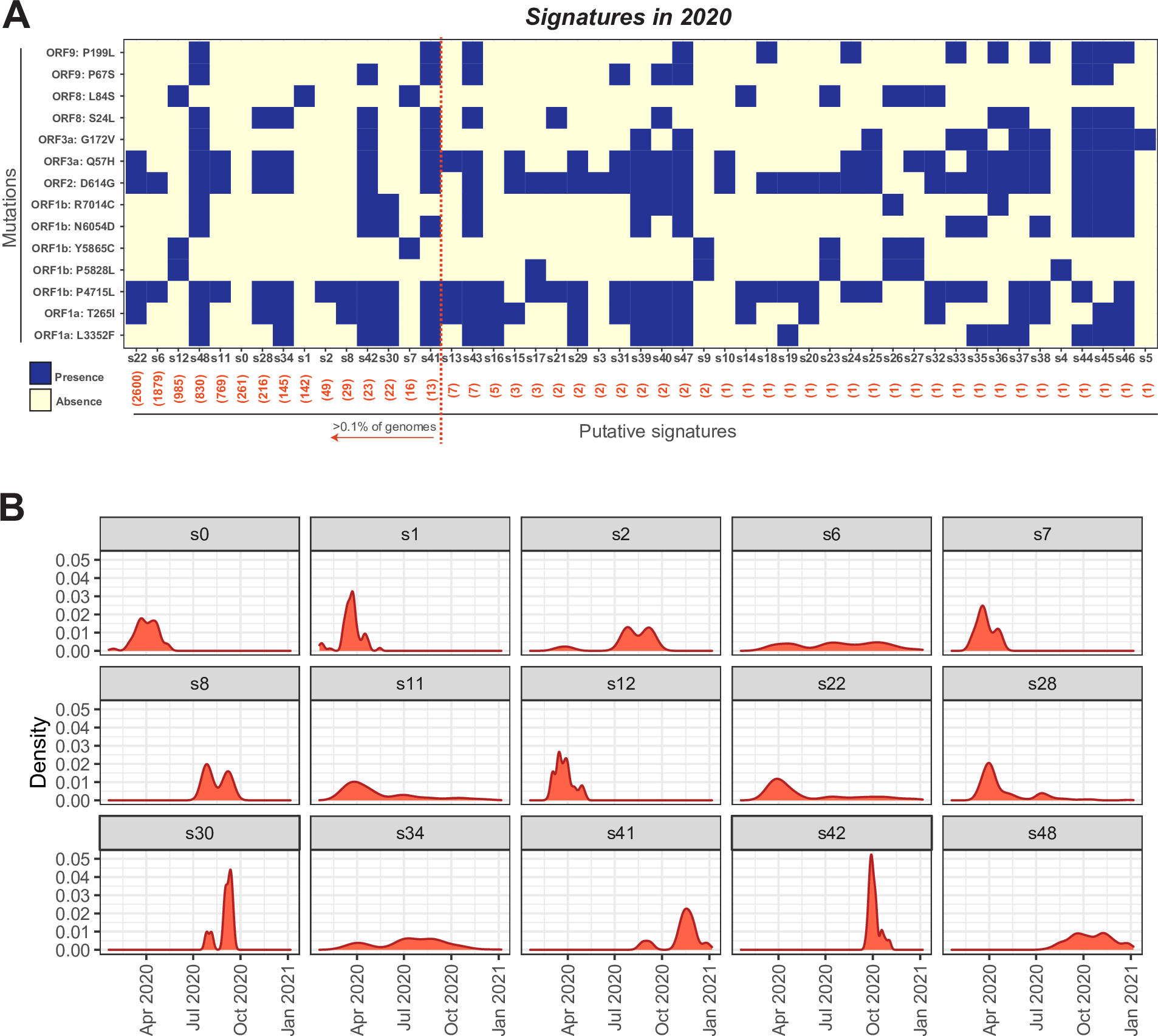

Supplement: S2 Fig — (A) Heatmap summarizing all the putative signatures (columns) built by the unique combination of dominant mutations (rows). Signatures are ranked from left to right by the sequence number they were found in (red labels below x axis). The first 15 signatures that are found in 0.1% of the sequences (>8) or more were considered for the mutational signature analysis. Presence or absence of mutation in each signature is denoted with blue or light yellow respectively. (B) Signatures that were found in more than 0.1% of the viral isolates in aggregate were further explored in the context of time from emergence till early 2021. Viral isolates that were profiled with those signatures (s0, s1-2, s6-8, s11-12, s22, s28, s34, s41-42, s48), were ordered by collection date in the columns of the heatmap from left to right. The heatmap visualizes the % occurrence (light yellow to dark blue scale) of each signature per collection date in the cohort of sequences. Column annotation (bottom of the heatmap) denotes the different quartiles (Q1-Q4) of calendar year 2020 (very few entries from Q1 of 2021 are shown) in which the sequences were collected. The non-variant SARS-CoV-2 (s0) is present primarily in the in Q1 up to mid-Q2 of 2020. While a diverse set of signatures appears in the USA from the start of the pandemic onward, subvariants currently circulating in the American population are variations of s48, s22 and s6 (with some variation per state). (TIF) [file pone.0255169.s002.tif]

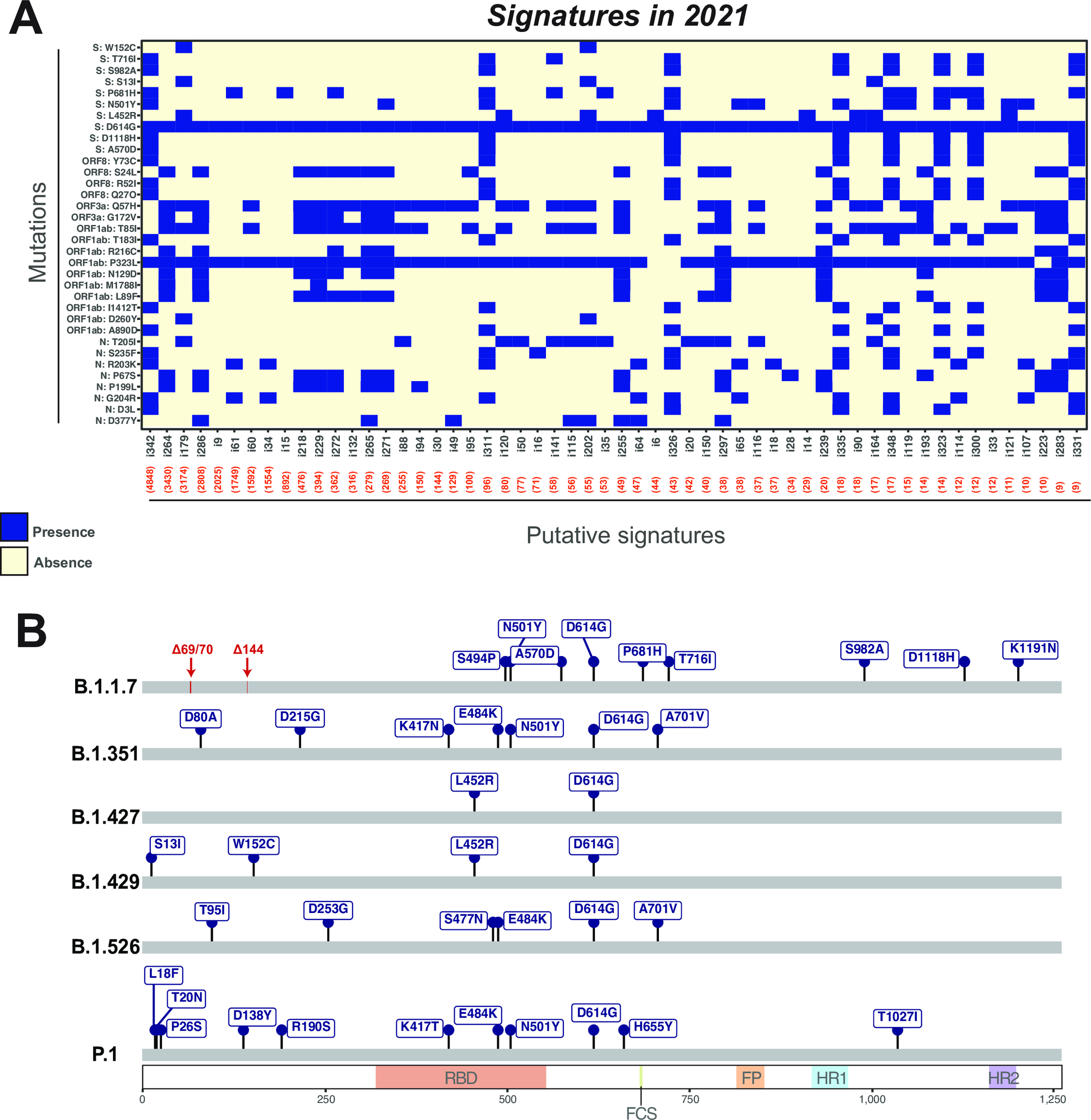

Supplement: S3 Fig — (A) Heatmap summarizing all the putative signatures (columns) built by the unique combination of dominant mutations (rows) in 2021. Signatures are ranked from left to right by the sequence number they were found in (red labels below x axis). The signature s48, which emerged in 2020, was found in 5838 genomes in 2021, which ranks it the top signature also for 2021. Furthermore, additional signatures of the same lineage (B.1.2) emerged, likely from s48 (S4 and S5 Figs) in 2021. Furthermore, variants of concern, such as B.1.1.7, are responsible for an introduction of new signatures in 2021 (S4 and S5 Figs). (B) Schematic representation of the defining spike mutations or deletions (ΔH69/V70 and Δ144) for the different variants of concern detected in this study (Fig 5). The different domains of the Spike protein noted are RBD/receptor binding domain (red), FCS/furin cleavage site (green), FP/fusion peptide (orange), HR1/heptad repeat region-1 (turquoise) and HR2/heptad repeat region-2 (violet). (TIF) [file pone.0255169.s003.tif]

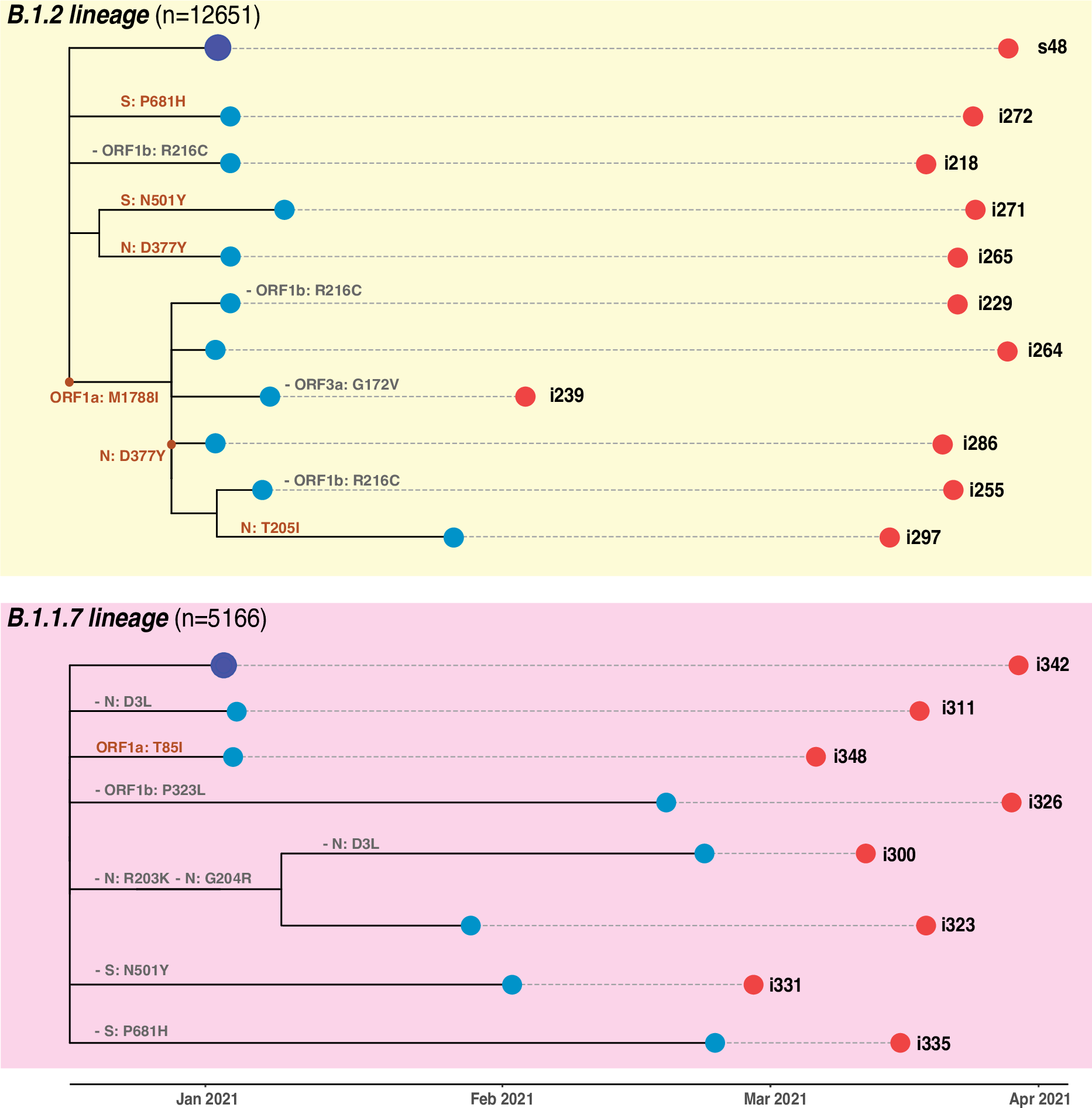

Supplement: S4 Fig — The signature s48, first detected in July 2020 (Fig 2C) is still present through the end of March 2021 and in fact is the top signature with the most genomes it has been found in (n = 5838). With the addition of several more mutations found predominant in 2021, we called unique profiles of co-occurring mutational signatures compiling 348 additional signatures (i1-i348; S3A Fig) distinct from the original Wuhan viral isolate (s0). Only the ones present in 0.01% genomes were considered for further analysis. In the first quartile of 2021 the lineage that profiles most of the genomes is B.1.2 (12651 genomes), followed by the lineage of the variant of concern B.1.1.7 (5166 genomes). Different signatures showed specificity for the aforementioned linages and therefore time-scaled phylogenetic trees were constructed separately for data collected in 2021, rooted to the respective variant genome. The founding genome for B.1.2 in the cohort of sequences was signature s48 (purple node and root) which is still present throughout the first quartile of 2021 and has further expanded to other signatures through loss (grey labels) and gain (red labels) of its original mutation profile (S2A Fig). For B.1.1.7 lineage, the first sequence detected, with the defining mutations of B.1.1.7 was the signature i342, which through mutation loss and gain has given additional related signatures. (TIF) [file pone.0255169.s004.tif]

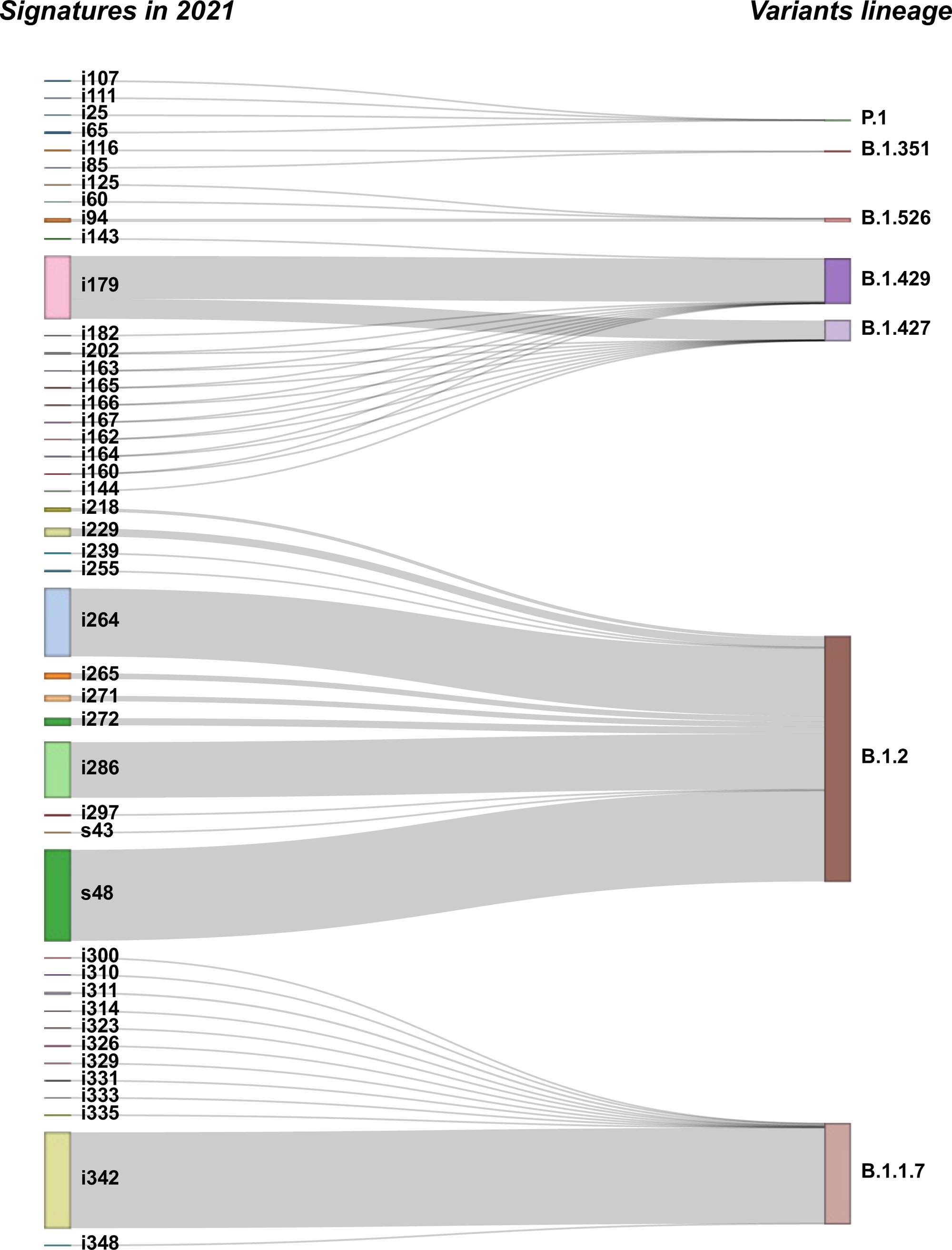

Supplement: S5 Fig — A number of variants of concern (P.1, B.1.351, B.1.526, B.1.429, B.1.427, B.1.1.7) were detected primarily in 2021, while genomes of the B.1.2 lineage remained abundant. This network shows the different versions of signatures detected in 2021 (>0.01% of the genomes) in lineages of the different variants of concern. The thicker the connection is, the higher the number of genomes were found for that lineage. The number of genomes profiled with the different signatures shown in here can be found in S3A Fig. (TIF) [file pone.0255169.s005.tif]
